# Genetic testing for rare pediatric lung disorders: The promise and the pitfalls

**DOI:** 10.1002/ped4.12179

**Published:** 2020-03-17

**Authors:** Lawrence M. Nogee

**Affiliations:** ^1^ Eudowood Division of Neonatology Department of Pediatrics Johns Hopkins University School of Medicine Baltimore Maryland USA

Genetic disorders of surfactant metabolism are rare causes of lung disease that arise from mutations in genes that encode proteins important in surfactant function and metabolism, and are associated with significant morbidity and mortality.^1^ Four different genes have been identified to date that can result in these disorders, with overlapping phenotypes that can range from severe neonatal respiratory distress syndrome to subacute interstitial lung disease in adults. Next generation DNA sequencing panels can simultaneously analyze multiple genes cost effectively, allowing for non‐invasive molecular diagnoses of these disorders, potentially avoiding the need for lung biopsies in critically ill patients.

Mutations in *SFTPC*, the gene encoding surfactant protein C (SP‐C), are associated with the most variable clinical course. The age of onset, severity and progression of lung disease in patients with *SFTPC* mutations varies greatly, even amongst family members with the same mutation, and the reasons for this variability are poorly understood. In last issue of Pediatric Investigation, Tang and colleagues report on five subjects with lung disease apparently due to *SFTPC* mutations identified using a multi‐gene panel, including one subject with features (diffuse alveolar hemorrhage and immune dysfunction) not previously associated with *SFTPC* mutations.^2^ Their findings thus potentially further expand the spectrum of disease associated with *SFTPC* mutations, and also raise provocative questions as to the incidence and prevalence of disease. Their diagnostic approach in using a panel that included over 1600 genes indicates the potential utility of such an approach in understanding how the contributions of multiple genes may lead to a certain phenotype, but also highlights the limitations of interpretations of genetic testing in deciding upon a diagnosis.

The subject in Tang’s report who also had immune dysfunction carried a well‐recognized pathogenic *SFTPC* mutation, a substitution of threonine for isoleucine in codon 73 (p.Ile73Thr). The data supporting that *SFTPC* p.Ile73Thr mutation is pathogenic are robust. There are multiple reports of patients with a consistent phenotype with this mutation , while it is absent in large population databases.^3^, ^4^, ^5^ The mutation both segregated with lung disease in familial cases and was identified in subjects with sporadic disease and *de novo* mutations. *In vitro* studies and its expression in genetically modified mice also support that it causes lung disease.^6^, ^7^ Immune dysfunction has not been part of the phenotype of other reported patients with this mutation. What might be different in the subject in this report?

The answer may lie in findings in other genes. In the supplementary data table, this subject was also found to have two variants in the gene (*LRBA*) encoding the LPS responsive beige‐like anchor protein, which were inferred to be on opposite alleles as the parents were also tested. LRBA deficiency is a recognized autosomal recessive cause of immune dysfunction, so if the identified *LRBA* variants are pathogenic then this child has two distinct genetic disorders.^8^ There is precedent for multiple genetic disorders in the same subject. In a large study using exome sequencing to identify novel genetic disorders, some subjects with had potentially significant findings in more than one gene.^9^, ^10^ Even if only one of the *LRBA* variants is pathogenic, it is possible that it contributed to the phenotype. Monoallelic mutations in another surfactant‐related gene, *ABCA3,* can modify the phenotype associated with *SFTPC* mutations.^11^

Potentially important variants in other genes were also identified the other subjects of this study. Of two other subjects who also had *SFTPC* p.Ile73Thr, one had multiple variants in surfactant related genes as well as in *MUC5B* — a gene in which a promoter variant influences the risk for pulmonary fibrosis and the other had a variant in *TSC2*, which can also be associated with lung disease. A subject with a different but previously reported as pathogenic *SFTPC* variant had two variants in a gene in which mutations cause primary ciliary dyskinesia. Thus, the broad examination of multiple genes that influence pulmonary and immune function may provide insights that could explain some of phenotypic variability due to *SFTPC* mutations.

This approach also illustrates challenges in interpretation of genetic results. One subject had the *SFTPC* mutation, p.Val39Leu. The apparent *de novo* occurrence of this mutation in a subject with sporadic disease and its segregation with disease in familial cases support that it may be disease‐causing.^12^ However, in a publically available database (https://gnomad.broadinstitute.org), the allele frequency for this variant in the East Asian population is 0.7% (133 in 18 870 alleles). While not common, this is also not exceeding rare and by extrapolation, almost 19.5 million Chinese individuals carry this mutation and thus could have or be at risk for *SFTPC* related lung disease! This subject also had a variant (p.Phe168Leu) in the gene *NKX2‐1* encoding Thyroid Transcription Factor 1 (which is important for surfactant protein gene expression) that has previously been associated with dominantly inherited lung disease.^13^ Thus, it is unclear whether this subject’s lung disease was due to the *NKX2‐1* mutation, the *SFTPC* mutation, or both acting in combination. Whether *SFTPC* p.Val39Leu is truly disease causing will require additional clinical and basic science studies.

Multigene panels are now readily available for diagnostic testing through multiple laboratories (www.ncbi.nlm.nih.gov/gtr), and the cost of sequencing the entire exome and genome continues to fall. The availability and increased utilization of such testing will increasing enable diagnosing rare disorders. However, as the study by Tang and colleagues indicates, the use of such approaches also presents challenges in establishing the correct diagnosis or diagnoses. It also illustrates the potential for understanding phenotypic variability due to multiple genetic variables. Sharing of data through publication of all potentially relevant findings such as in the current report will be essential for continued progress.

## CONFLICT OF INTEREST

None.
